# Ten years of external quality assessment (EQA) of *Neisseria gonorrhoeae* antimicrobial susceptibility testing in Europe elucidate high reliability of data

**DOI:** 10.1186/s12879-019-3900-z

**Published:** 2019-03-25

**Authors:** Michelle J. Cole, Nerteley Quaye, Susanne Jacobsson, Michaela Day, Elizabeth Fagan, Catherine Ison, Rachel Pitt, Shila Seaton, Neil Woodford, Angelika Stary, Sonja Pleininger, Tania Crucitti, Blaženka Hunjak, Panayiota Maikanti, Steen Hoffmann, Jelena Viktorova, Susanne Buder, Peter Kohl, Eva Tzelepi, Eirini Siatravani, Eszter Balla, Guðrún Svanborg Hauksdóttir, Lisa Rose, Paola Stefanelli, Anna Carannante, Gatis Pakarna, Francesca Mifsud, Rosann Zammit Cassar, Ineke Linde, Thea Bergheim, Martin Steinbakk, Beata Mlynarczyk-Bonikowska, Maria-José Borrego, Jill Shepherd, Peter Pavlik, Samo Jeverica, Julio Vazquez, Raquel Abad, Sabrina Weiss, Gianfranco Spiteri, Magnus Unemo

**Affiliations:** 1 Antimicrobial Resistance and Healthcare Associated Infections (AMRHAI) Reference Unit, National Infection Service, Public Health England, London, UK; 2 National Mycobacterium Reference Service, National Infection Service, Public Health England, London, UK; 3 WHO Collaborating Centre for Gonorrhoea and other STIs, Örebro University, Örebro, Sweden; 4 United Kingdom National External Quality Assessment Service (UK NEQAS), National Infection Service, Public Health England, London, UK; 5 Sexually Transmitted Bacteria Reference Unit (retired), Public Health England, London, UK; 6 Outpatients` Centre for the Diagnosis of Infectious Venero-Dermatological Diseases, Vienna, Austria; 7 National Reference Centre for Gonococci, Austrian Agency for Health and Food Safety, Vienna, Austria; 8 Institute of Tropical Medicine, Antwerp, Belgium; 9 Croatian Institute of Public Health, Zagreb, Croatia; 10 Microbiology Department, Nicosia General Hospital, Nicosia, Cyprus; 11 Department for Bacteria, Parasites and Fungi Infectious Diseases Preparedness, Statens Serum Institut, Copenhagen, Denmark; 12 Central Laboratory of Communicable Diseases, Tallinn, Estonia; 13 Laboratory for Gonococci, Vivantes Klinikum, South, Berlin, Germany; 14 National Reference Centre for N. gonorrhoeae, Laboratory of Bacteriology of the Hellenic Pasteur Institute, Athens, Greece; 15 Bacterial STI Reference Laboratory, National Public Health Institute, Budapest, Hungary; 16 Landspitali University Hospital, Reykjavik, Iceland; 17 National Gonococcal Reference Laboratory, St James’s Hospital, Dublin, Ireland; 18 Department of Infectious Diseases, Istituto Superiore di Sanità, Rome, Italy; 19 National Microbiology Reference Laboratory, Latvian Centre of Infectious Diseases, Riga East University Hospital, Riga, Latvia; 20 Bacteriology Laboratory, Mater Dei Hospital, Msida, Malta; 21 Streeklaboratorium/Bacteriologie, GGD Amsterdam, Amsterdam, The Netherlands; 22 Department of Medical Microbiology, Oslo University Hospital, Oslo, Norway; 23 Norwegian Institute of Public Health, Oslo, Norway; 24 Department of Diagnostics of Sexually Transmitted Diseases, Medical University of Warsaw, Warsaw, Poland; 25 Instituto Nacional de Saúde Doutor Ricardo Jorge, Lisbon, Portugal; 26 Scottish Bacterial Sexually Transmitted Infections Reference Laboratory, Royal Infirmary of Edinburgh, Edinburgh, UK; 27 Medirex a.s., Bratislava, Slovakia; 28 Institute of Microbiology and Immunology, Faculty of Medicine, University of Ljubljana, Ljubljana, Slovenia; 29 Reference Laboratory for Neisseria National Centre for Microbiology – Instituto de Salud Carlos III, Majadahonda, Spain; 30 Institute of Virology, Charite - Universitätsmedizin Berlin, Berlin, Germany; 31 European Centre for Disease Prevention and Control, Stockholm, Sweden

**Keywords:** Gonorrhoea, EUCAST, Euro-GASP, European Union (EU), European economic area (EEA)

## Abstract

**Background:**

Confidence in any diagnostic and antimicrobial susceptibility testing data is provided by appropriate and regular quality assurance (QA) procedures. In Europe, the European Gonococcal Antimicrobial Susceptibility Programme (Euro-GASP) has been monitoring the antimicrobial susceptibility in *Neisseria gonorrhoeae* since 2004. Euro-GASP includes an external quality assessment (EQA) scheme as an essential component for a quality-assured laboratory-based surveillance programme. Participation in the EQA scheme enables any problems with the performed antimicrobial susceptibility testing to be identified and addressed, feeds into the curricula of laboratory training organised by the Euro-GASP network, and assesses the capacity of individual laboratories to detect emerging new, rare and increasing antimicrobial resistance phenotypes. Participant performance in the Euro-GASP EQA scheme over a 10 year period (2007 to 2016, no EQA in 2013) was evaluated.

**Methods:**

Antimicrobial susceptibility category and MIC results from the first 5 years (2007–2011) of the Euro-GASP EQA were compared with the latter 5 years (2012–2016). These time periods were selected to assess the impact of the 2012 European Union case definitions for the reporting of antimicrobial susceptibility.

**Results:**

Antimicrobial susceptibility category agreement in each year was ≥91%. Discrepancies in susceptibility categories were generally because the MICs for EQA panel isolates were on or very close to the susceptibility or resistance breakpoints. A high proportion of isolates tested over the 10 years were within one (≥90%) or two (≥97%) MIC log_2_ dilutions of the modal MIC, respectively. The most common method used was Etest on GC agar base. There was a shift to using breakpoints published by the European Committee on Antimicrobial Susceptibility Testing (EUCAST) in the latter 5 years, however overall impact on the validity of results was limited, as the percentage categorical agreement and MIC concordance changed very little between the two five-year periods.

**Conclusions:**

The high level of comparability of results in this EQA scheme indicates that high quality data are produced by the Euro-GASP participants and gives confidence in susceptibility and resistance data generated by laboratories performing decentralised testing.

## Background

Gonorrhoea is the second most common bacterial sexually transmitted infection (STI) worldwide, with a global estimate of 78 million new cases among adults in 2012 [1]. If untreated, gonorrhoea can result in complications and sequelae such as pelvic inflammatory disease, ectopic pregnancy and infertility [2]. In the absence of an effective vaccine, antimicrobial treatment along with appropriate prevention, diagnostics and surveillance, is the mainstay in the clinical and public health management of gonorrhoea and prevention of these complications. However, due to the emergence and spread of antimicrobial resistance in the causative agent, *Neisseria gonorrhoeae,* most previously used therapeutic agents can no longer be recommended for first-line treatment [3]. Dual antimicrobial therapy, mainly ceftriaxone 250–500 mg plus azithromycin 1–2 g, is the current recommended empirical first-line treatment for gonorrhoea in many countries [4]. As strongly emphasized in the WHO global action plan [5] and the European response plan [6] to control multidrug-resistant *N. gonorrhoeae*, enhanced worldwide, quality-assured surveillance of gonococcal antimicrobial susceptibility is crucial in order to ensure the effectiveness of the recommended empiric treatment, to monitor antimicrobial resistance trends, and to identify new emerging resistance.

In general, confidence in any diagnostic and antimicrobial susceptibility testing data is provided by appropriate and regular quality assurance (QA) procedures. These include validations of testing methods used, internal quality controls, and quality assessments such as internal quality assessment and, importantly, external quality assessment (EQA). In Europe, the European Gonococcal Antimicrobial Susceptibility Programme (Euro-GASP) has been monitoring the antimicrobial susceptibility in *N. gonorrhoeae* since 2004 [7–11]. Isolates are tested either centrally or via a decentralised testing model where antimicrobial susceptibility testing is performed in laboratories in participating countries after fulfilling set quality criteria. Criteria include acceptable performance in the EQA and good comparability between the laboratories own national susceptibility testing data and susceptibility data generated by centralised susceptibility testing [11]. Euro-GASP includes an EQA scheme as an essential component for a quality-assured laboratory-based surveillance programme [12]. This EQA scheme aims to ensure high-quality, accurate and comparable susceptibility data between and within testing laboratories. Furthermore, successful performance in the EQA is one of the quality criteria (introduced in 2010) required for Euro-GASP to include susceptibility data generated by laboratories performing decentralised testing [11]. Participation in the EQA scheme enables any problems with the performed antimicrobial susceptibility testing to be identified and addressed, feeds into the curricula of laboratory training organised by the Euro-GASP network, and assesses the capacity of individual laboratories to detect emerging new, rare and increasing antimicrobial resistance phenotypes.

The first Euro-GASP EQA gonococcal strain panel was distributed to the newly created Euro-GASP network in 2003 [13], before the first sentinel susceptibility study in 2004 [9]. The results showed an overall susceptibility category agreement of only 70% and a crucial need for enhanced standardisation of the susceptibility testing methods used in Europe. In 2007, the Euro-GASP EQA scheme was re-established and subsequently run until 2009 as part of the European Surveillance of Sexually Transmitted Infections (ESSTI) programme [14]. Since 2010, the EQA has been co-ordinated by the European Centre for Disease Prevention and Control (ECDC).

The aims of the present study were to evaluate the performance of the Euro-GASP EQA over a 10-year period (2007 to 2016) and to compare the results of the first 5 years (2007–2011) with the latter 5 years (2012–2016; no EQA in 2013) in order to assess whether Euro-GASP data provide a high-quality and valid picture of gonococcal antimicrobial resistance in the European Union/European Economic Area (EU/EEA), i.e. on which treatment recommendations can be based. These time periods were selected to additionally assess the impact of the 2012 European Union case definitions for the reporting of antimicrobial susceptibility (http://eur-lex.europa.eu/LexUriServ/LexUriServ.do?uri=OJ:L:2012:262:0001:0057:EN:PDF).

## Methods

### EQA panel and antimicrobial susceptibility testing methods

The EQA gonococcal strain panels were selected by Public Health England (PHE) and Örebro University Hospital, Sweden. From 2007 to 2009, the EQA was run annually, consisted of 30 cultures of *N. gonorrhoeae* (10 strains in triplicate to measure intra-laboratory reproducibility), and was distributed by PHE to 19 participating laboratories in 2007 and 2008, and 16 laboratories in 2009. Since 2010, the United Kingdom National External Quality Assessment Service (UK NEQAS) has distributed the EQA panels. The EQA panels from 2010 to 2016 (no EQA was performed in 2013) consisted of 65 gonococcal isolates, some in duplicate or triplicate. In 2010, 15 isolates were distributed in two EQAs; ten isolates in March (19 laboratories) and five in October (20 laboratories). In 2011, two panels of five isolates each were distributed in February (20 laboratories) and October (21 laboratories). Ten isolates were distributed in one panel in 2012 (22 laboratories), 2014 (21 laboratories), 2015 (26 laboratories), and 2016 (27 laboratories). The gonococcal strains in the EQA panels demonstrated a range of antimicrobial susceptibility profiles to therapeutic agents. The gonococci were selected from clinical isolates and a panel of well characterised strains, including current WHO reference strains [15]. Over the 10 years, 46 different strains were included in the EQAs; ten of these strains were included in more than one distribution (four strains in three distributions, two strains in two and four distributions (four strains in total), one strain in five and another in seven distributions). Of the ten different strains, eight (WHO F, G, K, L, M, N, O, P) are incorporated in the WHO *N. gonorrhoeae* control panel [15] and were included a total of 29 times.

The laboratories participating in the EQA scheme were requested to test the isolates using their own routine antimicrobial susceptibility testing methodology and standard operating procedures, against a panel of therapeutically relevant antimicrobial agents, ideally ceftriaxone, cefixime (included since 2010), azithromycin, ciprofloxacin, spectinomycin, and gentamicin (included since 2010). Penicillinase production (β-lactamase testing) was also monitored where performed. The antimicrobial susceptibility testing methodologies, including media used, and the guidelines/breakpoints used for each antimicrobial agent were requested. Data on the clinical breakpoints (interpretative criteria) used by each laboratory for each individual agent was available until 2012. Post 2012, data were collected on adherence to published breakpoints. For each isolate tested, minimum inhibitory concentrations (MICs) (mg/L) or zones of inhibition (mm), as well as the susceptibility category (susceptible (S), intermediate susceptible (I), or resistant (R)) were reported. Results were reported directly to PHE until 2012 and to UK NEQAS from 2014.

### Analysis and interpretation of the results

For the analysis, Etest (or more rarely other MIC gradient strip tests such as the Oxoid M.I.C.Evaluator strips or the Liofilchem MIC Test Strips) whole MIC log_2_ dilutions were used. The minimum, maximum and modal MIC of each strain was established. To avoid relying upon one set of MIC results from the laboratory that selected the isolates, the modal MIC was used as the ‘expected’ MIC. The number of MICs within one MIC log_2_ dilution of the modal MIC (essential agreement), as well as number of MICs within two and > 2 MIC log_2_ dilutions of the modal MIC for each strain was established for all years and for each five-year period (2007–2011 and 2012–2016). The MICs for the eight WHO strains (total of 29 appearances in the EQA) were analysed to assess comparability, consistency and performance of participating laboratories over the 10 years.

The consensus antimicrobial susceptibility category (S, I or R) was assigned for each strain and antimicrobial agent tested and from all isolates in the triplicate or duplicate sets, irrespective of breakpoint criteria used. The overall percentage susceptibility category agreement for each antimicrobial agent was established by calculating the average of each strain percentage concordance. The susceptibility category agreement for each year was the average of each of the concordances for each agent. Due to the confidential nature of the EQA, all results were aggregated by year and antimicrobial.

### Troubleshooting

Any laboratory that reported more than 5% of strains with MICs > 2 MIC log_2_ dilutions from the modal MIC was contacted to identify problems with contamination, reagents, testing and interpretation.

## Results

### Antimicrobial susceptibility testing methods

The Etest (or more rarely other MIC gradient strip tests) was the most common antimicrobial susceptibility testing method, used in 55.0% of laboratories in 2007–2011 and 76.8% in 2012–2016 (Table 1). The use of disk diffusion as the sole method decreased from 21.4% in 2007–2011 to 3.2% of laboratories in 2012–2016, when most laboratories had replaced their disk diffusion methods with Etest due to its better performance in the EQAs in general, and the recommendations by the Euro-GASP. The most frequently used agar media or agar base for the antimicrobial susceptibility testing was GC agar base (63.4% of laboratories in 2007–2011 and 53.7% in 2012–2016). An increased use of non-selective Thayer-Martin and non-specified agar/s accounted for the reduction in the use of GC agar over the two time periods. Guidelines/breakpoints from the Clinical and Laboratory Standards Institute (CLSI) [16] were adhered to most frequently in the first five-year time period (69.8%), whereas breakpoints from the European Committee on Antimicrobial Susceptibility testing (EUCAST) [17] were the most common in the second five-year time period (increased from 6.5 to 65.3%) (Table 1). Most variation in the applied SIR breakpoints was observed for azithromycin (Table 2) with some harmonisation to EUCAST breakpoints observed from 2007 (0%) to 2012 (42.9%). However, many different breakpoints were used also for cefixime, ceftriaxone, ciprofloxacin and spectinomycin (Table 3).

**Table 1:** Details of antimicrobial susceptibility testing methods used in the European gonococcal External Quality Assessments (EQAs)

		2007–2011 (n=131^a^)		2012–2016 (n=95^a^; no EQA in 2013)	
		No.	%	No.	%
Antimicrobial susceptibility test	Etest^b^	72	55.0	73	76.8
	Agar dilution	26	19.8	13	13.7
	Disk diffusion	28	21.4	3	3.2
	Disk diffusion and Etests	5	3.8	6	6.3
Culture media/base	GC agar base	83	63.4	51	53.7
	Chocolatised blood agar	33	25.2	23	24.2
	Diagnostic sensitivity (DST) agar	7	5.3	5	5.3
	Thayer-Martin agar (non-selective)	5	3.8	10	10.5
	Blood agar base	2	1.5	1	1.1
	None specified	1	0.8	5	5.3
Guidelines/breakpoints ^c^	CLSI	97	69.8	23	24.2
	None specified	17	12.2	1	1.1
	GRASP – United Kingdom	7	5.0	4	4.2
	CACFM – France	3	2.2	4	4.2
	SRGA – Sweden	5	3.6	0	0.0
	EUCAST	9	6.5	62^d^	65.3
	WHO	1	0.7	1	1.1

*CLSI* Clinical and Laboratory Standards Institute [16], *GRASP* Gonococcal Resistance to Antimicrobials Surveillance Programme [34], *CA-SFM* Committee of the French Society for Microbiology (http://www.sfm-microbiologie.org), *SRGA* Swedish Reference Group for Antibiotics (no longer operational), *EUCAST* European Committee on Antimicrobial Susceptibility testing [17], *WHO* World Health Organization

^a^Some methods and guidelines changed throughout the time periods for some laboratories. Thus, analysis of the comparison of methods was performed using each laboratory for each year to give a total of 131 comparisons for 2007–2012 and 95 for 2012–2016

^b^During recent years, some countries have also used other MIC gradient strip tests

^c^*n* = 139 for guidelines/breakpoints as some laboratories used more than one guidance on methodology/breakpoints over the time period

^d^Includes one laboratory that also used BSAC disk diffusion breakpoints for azithromycin

**Table 2:** Different MIC breakpoints for azithromycin used in the European gonococcal External Quality Assessments (EQAs) from 2007 to 2012

No. of laboratories* (*n* = 104)	No. of laboratories 2007 (*n* = 12)	No. of laboratories 2012 (*n* = 14)	Azithromycin MIC breakpoints (mg/L)		
			S ≤	I	R >
31 (29.8%)	4 (33.3%)	4 (28.6%)	0.5	–	0.5
29 (27.9%)	0	6 (42.9%)	0.25^a^	0.5^a^	0.5^a^
11 (10.6%)	2 (16.7%)	1 (7.1%)	1	–	1^b^
7 (6.7%)	2 (16.7%)	0	0.5	1	1
6 (5.8%)	1 (8.3%)	0	2	–	–
6 (5.8%)	0	2 (14.3%)	0.25	–	0.25
1 (1.0%)	0	0	0.125	0.25–0.5	0.5
1 (1.0%)	0	0	0.125	0.25–1	1
1 (1.0%)	1 (8.3%)	0	0.25	0.5–1	1
1 (1.0%)	1 (8.3%)	0	4		
10 (9.6%)	1 (8.3%)	1 (7.1%)	None given		

Note: Detailed interpretative MIC data was only available until 2012. Adherence to published breakpoints collected post-2012

*Includes total number of participants at each distribution, i.e. the same laboratory will be counted at each distribution

^a^Current EUCAST breakpoints

^b^US GISP alert value MIC (https://www.cdc.gov/std/gisp/GISP-Protocol-May-2016.pdf)

**Table 3:** Different MIC and zone diameter breakpoints for cefixime, ceftriaxone, spectinomycin and ciprofloxacin used in the European gonococcal External Quality Assessments (EQAs) from 2007 to 2012

Antimicrobial agent	MIC (mg/L) and zone diameter (mm) breakpoints		
	S ≤	I	R >
Cefixime	0.06	≥0.12	–
	0.12	≥0.25	–
	0.12^a^	–	0.12^a^
	0.25^b^	–	–
	≥31^c^	–	–
Ceftriaxone	0.06	≥0.12	–
	0.12	≥0.25	–
	0.12^a^	–	0.12^a^
	0.25^b^	–	–
	≥35^c^	–	–
Spectinomycin	64^a^	–	64^a^
	32	–	32
	32^b^	64^b^	64^b^
	≥18^c^	15 – 17^c^	≤14^c^
Ciprofloxacin	0.03	0.06 - 0.5	0.5
	0.03	–	0.03
	0.03^a^	–	0.06^a^
	0.12	–	0.25
	0.06^b^	0.12 - 0.5^a^	0.5^b^
	≥41^c^	28 – 40^c^	≤27^c^

Note: Detailed interpretative MIC data was only available until 2012. Adherence to published breakpoints collected post-2012

^a^Current EUCAST breakpoints [17]

^b^Current CLSI breakpoints [16]

^c^Zone diameter breakpoints (mm)

All centres using the disk diffusion method referred to the antibiotic contents recommended by the CLSI [16], with exception of the azithromycin (15 μg) and gentamicin (10 μg) disks for which CLSI does not have any recommendations. The majority of the centres using disk diffusion method also adhered to the recommended CLSI zone diameter breakpoints [16], again with the exception of the azithromycin resistance zone diameter breakpoints of ≤25 mm, ≤27 mm (BSAC) and ≤ 30 mm and gentamicin which has no defined SIR breakpoints.

### Antimicrobial susceptibility category agreement

**Fig. 1:**
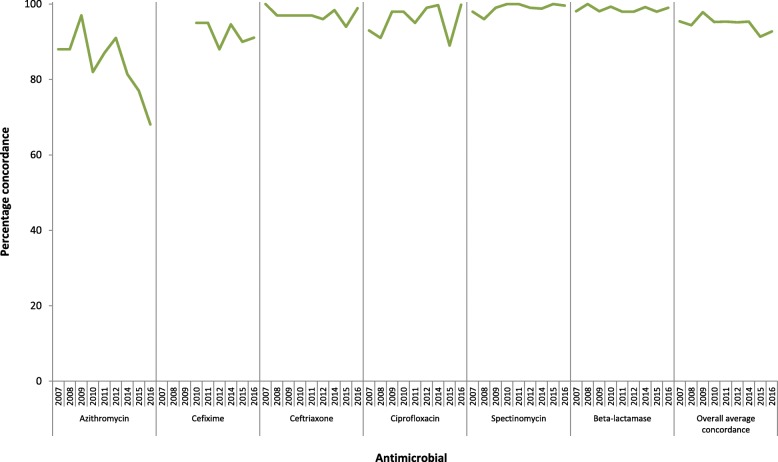
Overall antimicrobial susceptibility category agreement in the European gonococcal External Quality Assessments (EQAs), 2007–2016 (no EQA in 2013). Note: Cefixime was not tested before 2010

The overall antimicrobial susceptibility category agreement was consistently very high for spectinomycin (mean: 99.0%; range over the years: 96–100%), β-lactamase testing (98.6%; 98–100%) and ceftriaxone (97.2%; 94–100%). The concordance was also high for ciprofloxacin (95.9%; 89–100%) and cefixime (92.3%; 88–95%). However, for azithromycin the concordance was lower and fluctuated substantially over the years (84.3%; 68–97%) (Fig. 1). The lowest concordance for azithromycin (68%) was noted in 2016. Consensus antimicrobial susceptibility categories were not assigned for gentamicin as no international organisation has stated any SIR breakpoints for interpretation of results.

The susceptibility category agreement was either the same or higher for agar dilution compared with Etest, with ceftriaxone (2012–2015) being the exception. In general, the susceptibility category agreement over the two time periods of 2007–2011 and 2012–2015 was lowest for azithromycin (88 and 83%, respectively) and highest for spectinomycin (99%) (Table 4).

**Table 4:** Overall concordance (%) of susceptibility category agreement for all EQA distributions and methods 2007–2011 and 2012–2015 (no EQA in 2013)

	2007–2011			2012–2015 (no EQA in 2013)^a^		
	All methods (*n* = 131)^b^	Etest (*n* = 72)^c^	Agar dilution (*n* = 26)	All methods (*n* = 66)^b^	Etest (*n* = 52)^c^	Agar dilution (*n* = 11)
Azithromycin	88	85	97	83	80	92
Cefixime^d^	95	93	99	91	91	91
Ceftriaxone	98	97	98	96	97	96
Ciprofloxacin	95	96	97	96	96	96
Spectinomycin	99	99	99	99	99	100

^a^2016 results not included as only two laboratories performed agar dilution in 2016 so concordance was not calculated

^b^Includes disk diffusion results

^c^Some countries have also used other MIC gradient strip tests

^d^Not tested prior to 2010

### Concordance of MIC

Overall, high proportions of the reported MICs of each antimicrobial agent were within one MIC log_2_ dilution (+/− two-fold variation) of the modal MIC, that is, gentamicin (95%), cefixime (93%), ciprofloxacin (92%), spectinomycin (91%), azithromycin (89%), and ceftriaxone (85%) (Table 5). Ninety percent of the total MICs were also within one MIC log_2_ dilution of the modal MIC during the two five-year periods (2007–2011 and 2012–2016), with exception of azithromycin (88% in 2012–2016) and ceftriaxone (84% in 2007–2011 and 88% in 2012–2016). In both time periods and for all antimicrobial agents combined, ≥97% of isolates were within two MIC log_2_ dilutions (+/− four-fold variation) of the modal MIC. The overall concordance did not change over the two five-year time periods (Table 5).

**Table 5:** MIC concordance and variation from the modal MIC in the European gonococcal External Quality Assessments (EQAs), 2007–2016 (no EQA in 2013)

		Azithromycin		Cefixime^a^		Ceftriaxone		Ciprofloxacin		Gentamicin^a^		Spectinomycin		Total	
		No.	%	No.	%	No.	%	No.	%	No.	%	No.	%	No.	%
2007–2011	Within 1 MIC log_2_ dilution^b^	1284	90	301	94	1331	84	1502	94	233	99	1038	91	5690	90
	Within 2 MIC log_2_ dilutions	123	99	13	98	172	95	80	99	2	100	76	98	466	98
	> 2 MIC log_2_ dilutions	19	1	6	2	76	5	24	1	0	0	26	2	151	2
	Total no. of isolates with MIC data	1426		320		1579		1606		235		1140		6307	
2012–2016	Within 1 MIC log_2_ dilution^b^	701	88	757	93	728	88	728	90	482	93	660	92	4056	91
	Within 2 MIC log_2_ dilutions	65	96	33	97	77	97	45	96	30	99	45	98	295	97
	> 2 MIC log_2_ dilutions	28	4	22	3	27	3	36	4	4	1	11	2	128	3
	Total no. of isolates with MIC data	794		812		832		809		516		716		4479	
All years	Within 1 MIC log_2_ dilution^b^	1985	89	1058	93	2059	85	2230	92	715	95	1698	91	9745	90
	Within 2 MIC log_2_ dilutions	188	98	46	98	249	96	125	98	32	99	121	98	761	97
	> 2 MIC log_2_ dilutions	47	2	28	2	103	4	60	2	4	1	37	2	279	3
	Total no. of isolates with MIC data	2220		1132		2411		2415		751		1856		10,785	

^a^Cefixime and gentamicin were not included in the 2007–2009 External Quality Assessment distributions

^b^Essential agreement

**Fig. 2:**
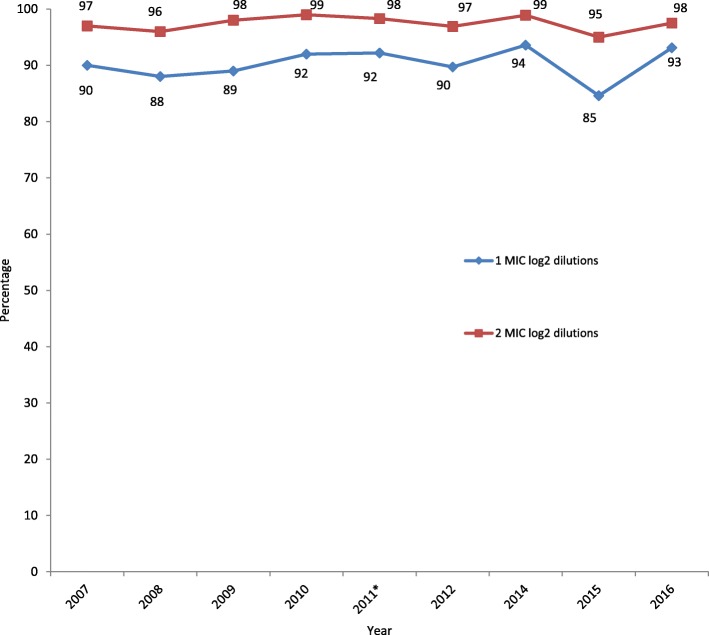
MIC concordance within one and two MIC log_2_ dilutions of the modal MIC in the European gonococcal External Quality Assessments (EQAs), 2007–2016 (no EQA in 2013). *contains isolates from 2010 (11–15) and 2011 (1–15)

The overall MIC concordances for each EQA distribution were ≥ 85% (mean: 90.7%; range: 85–94%) and ≥ 95% (97.6%; 95–99%) within one and two MIC log_2_ dilutions of the modal MIC, respectively (Fig. 2). Modal MICs for the eight WHO strains [15], used 29 times throughout the years, varied by one MIC log_2_ dilution, except for WHO M and ciprofloxacin which varied from 1 to 4 mg/L between two distributions, but the category remained the same, resistant. All susceptibility categories were identical except for WHO K and cefixime between two distributions (S and R), however the modal MIC was the same at 0.25 mg/L.

### Troubleshooting

More than 5% variation from the modal MIC was mainly identified in laboratories that did not yet participate in Euro-GASP decentralised testing. Issues identified and mostly corrected included use of media suboptimal for *N. gonorrhoeae* antimicrobial susceptibility testing, the use of MIC gradient strip tests other than Etests (BioMerieux), suboptimal reading of the MIC gradient strips, mis-interpretation of the colour change with nitrocefin, contamination and transcription errors.

## Discussion

An increasing number of laboratories participated in the ECDC Euro-GASP EQA; 27 in 2016 compared with 16 in 2009. Etest was the most common methodology and GC agar base the most frequently used agar. In the last 5 years, there was a marked shift among participants to the use of EUCAST [17] breakpoints from the CLSI [16] breakpoints, most likely influenced by Euro-GASP and the publication of the EU case definitions in August 2012 (http://eur-lex.europa.eu/LexUriServ/LexUriServ.do?uri=OJ:L:2012:262:0001:0057:EN:PDF), which include definitions of antimicrobial resistance and state that EUCAST clinical breakpoints [17, 18] should be adhered to. However, the lack of a recommended methodology for *N. gonorrhoeae* susceptibility testing by EUCAST might result in some laboratories continuing to use the CLSI breakpoints [16], which are inherently linked to the CLSI methodology, which may impact the interpretation. The harmonisation of antimicrobial susceptibility testing methods in the latter five-year period (2012–2016) in Europe is a substantial improvement compared with when the first EQA was performed and generally no common methods were used [13]. However, even though methods and SIR breakpoints have increasingly been harmonised, the overall impact on the validity of results has been very limited since 2007, as the percentage susceptibility category agreement and MIC concordances changed very little between the two five-year periods. Nevertheless, Euro-GASP will work together with EUCAST to establish best practice so laboratories across Europe can use standardised antimicrobial susceptibility testing methods for *N. gonorrhoeae*.

The United States Food and Drug Administration (FDA) recommends that essential agreement (within one MIC log_2_ dilution of the modal MIC) and antimicrobial susceptibility category agreement should be at a minimum 90% for each antimicrobial agent [19]. In the present study, the overall concordance of antimicrobial susceptibility categories in each year reached this target (≥91%), which is a major improvement compared with the first EQA performed in 2003 (> 70%). This demonstrates an improved level of comparability of antimicrobial susceptibility results, despite the diversity of methods. In future Euro-GASP EQAs, categorical agreement using a known number of non-susceptible strains and the number of very major, major and minor errors will be established, as recommended by CLSI [20]. The comparability of Etest and agar dilution observed in this study, has also been observed elsewhere, particularly for cephalosporins [21–23]. MIC differences in our study could be due to agar media and inoculum size differences as established previously [24, 25], along with varying levels of comparability between different disk diffusion methods and agar dilution and/or Etests [26–30]. Identified agar media differences in this study (data not shown) in general agree with data presented from other studies, including that cephalosporin MICs were generally slightly higher from agar dilution with GC agar compared with Etests [21–23], MICs from Etests with chocolate agar were higher [31], as well as differing MIC variation depending upon which media was used for agar dilution [32, 33]. It was suggested by Liao et al. [31] that laboratories should adhere to CLSI media recommendations (GC agar base with 1% growth supplement) [16], however the lack of commercial, off-the-shelf options of this medium makes this challenging for laboratories who do not have in-house agar-plate pouring facilities.

Discrepancies in susceptibility categories were generally because the MICs for EQA panel isolates were on or very close to the breakpoints, particularly for azithromycin in 2016 (68%), as well as for ciprofloxacin in 2014 (89%) when a triplicate was composed of isolates with MICs exactly on the resistance breakpoint. The use of triplicates/duplicates allows laboratories to investigate their own intra-laboratory concordance. However, if strains with MICs exactly on or very close to a breakpoint are selected, the overall category agreement for that antimicrobial agent can be highly affected. For this reason comparisons over time are difficult, so the inclusion of the same strains over the years, as achieved in this EQA and with comparable results, is an important aspect to consider when analysing EQA performance. Even though strains with MICs close to a breakpoint can impact on susceptibility category agreement, they are clinically relevant, effectively challenge the antimicrobial susceptibility testing in participating laboratories, and should be included in EQAs. However, when interpreting susceptibility category results it is important to consider the actual MIC for individual strains in respect to patient management and the MIC distribution for isolates contributing to susceptibility surveillance data, so isolates near or on breakpoints can be identified and appropriate caution issued. Differences in breakpoints will also impact on susceptibility category agreement, for example ten different breakpoint schemes for azithromycin were used by EQA participants from 2007 to 2012, and the breakpoints for cefixime were less harmonised during earlier years, which may have contributed to the lower concordance in 2012 (88%).

High comparability of results was also demonstrated by the high proportion of isolates tested over the 10 years that were within one (≥90%) MIC log_2_ dilution and two MIC log_2_ dilutions (≥97%) of the modal MIC. The overall lower MIC concordance for ceftriaxone (85%) may be due to the smaller dilution scales due to the mostly lower ceftriaxone MICs and requiring more precision, e.g. at 0.004, 0.008 and 0.016 mg/L, as compared with other antimicrobial agents with higher MICs in the dilution scales such as 4, 8, 16 mg/L. The lower MIC concordance for azithromycin (89%) was likely affected by the fact that MIC testing for azithromycin is very sensitive to minor differences in methodologies, in particular the medium used and pH (which is affected by the concentration of CO_2_), as has been demonstrated previously [33, 34]. Full concordance in antimicrobial susceptibility categories and MICs will likely never be possible, due to the inherent inter-assay variation of any testing method, and particularly not before there is a complete harmonisation of antimicrobial susceptibility testing methods.

The use of the same WHO strains over the years allowed the measurement of variability over time, which was shown to be very low in this EQA. The present study has shown that the inter-laboratory reproducibility was high amongst participating laboratories, comparable in different distributions and years, and the use of standardised quality control strains [15] allows improved comparison of results over time and between as well as within laboratories.

The Euro-GASP EQA revealed high levels of competence and capability in recovering and testing strains of unknown phenotype. The high level of comparability over the 10 years of the EQA indicates that high quality data are produced by the Euro-GASP participants and gives confidence in decentralised testing and comparison of antimicrobial susceptibility surveillance data in the EU/EEA. The results from this EQA compared well with similar national schemes in Canada (> 90% for MIC and interpretation concordance) [35], India (82% interpretation concordance) [36] and Australia (3.1% error rate in respect to penicillin MICs) [32], even though the Euro-GASP EQA is regional with many different participating countries, which by default means more variability in methodologies. A quality control comparison programme for the Latin America and the Caribbean GASP region recently reported that most participants had acceptable results and the impact of the different methods on the results was also highlighted [37].

It should be noted that the Euro-GASP laboratories are frequently experienced national reference laboratories with a high level of expertise and access to training and advice from the Euro-GASP coordinators. In contrast, the global GASP coordinated by the WHO includes antimicrobial susceptibility data from both experienced as well as less experienced laboratories. It would be exceedingly valuable to implement a global EQA scheme, particularly in regions not participating in existing schemes, to monitor and support comparability of antimicrobial susceptibility surveillance data from different countries and laboratories globally. In addition, the use of a global EQA could support primary diagnostic laboratories that perform antimicrobial susceptibility testing for patient management and local surveillance studies to ensure adequate quality. The crucial need for this was illustrated in a national survey in the United Kingdom [38], where low levels of QA in gonococcal antimicrobial susceptibility procedures were identified. Confidence in the reporting of patient related antimicrobial susceptibility results is essential to avoid administering inappropriate treatment. A global EQA would additionally allow the global dissemination of important reference strains for QA and clinical strains with interesting/emerging resistance profiles or diagnostically challenging characteristics, and provide a further opportunity for laboratories to achieve accreditation standards.

## Conclusions

Gonorrhoea remains a public health concern because of the increasing incidence and the threat of multidrug-resistant *N. gonorrhoeae*. Strengthening surveillance of gonococcal antimicrobial susceptibility is imperative worldwide and, in Europe, Euro-GASP has been expanding annually. The high level of QA of the data from Euro-GASP and other similar surveillance programmes is essential in order to identify novel emerging resistance, appropriately monitor antimicrobial resistance trends and to ensure national and international gonorrhoea treatment guidelines are updated based on high quality and valid antimicrobial susceptibility data.

## Abbreviations

CLSI: Clinical and Laboratory Standards Institute
ECDC: European Centre for Disease Prevention and Control
EEA: European Economic Area
EQA: External quality assessment
ESSTI: European Surveillance of Sexually Transmitted Infections
EU: European Union
EUCAST: European Committee on Antimicrobial Susceptibility testing
Euro-GASP: European Gonococcal Antimicrobial Surveillance Programme
FDA: Food and Drug Administration
MIC: Minimum inhibitory concentration
PHE: Public Health England
QA: Quality assurance
S, I, R: Susceptible, intermediate susceptible, resistant
STI: Sexually transmitted infection
UK NEQAS: United Kingdom National External Quality Assessment Service
US: United States
WHO: World Health Organization
